# Clinical Significance and Prognostic Value of the Maximum Standardized Uptake Value of ^18^F-Flurodeoxyglucose Positron Emission Tomography–Computed Tomography in Colorectal Cancer

**DOI:** 10.3389/fonc.2021.741612

**Published:** 2021-12-09

**Authors:** Yi-xin Yin, Ming-zhi Xie, Xin-qiang Liang, Meng-ling Ye, Ji-lin Li, Bang-li Hu

**Affiliations:** ^1^ Department of Research, Guangxi Medical University Cancer Hospital, Nanning, China; ^2^ Department of Chemotherapy, Guangxi Medical University Cancer Hospital, Nanning, China

**Keywords:** colorectal cancer, ^18^FDG-PET/CT, SUVmax, KRAS, Ki-67, prognosis

## Abstract

**Background:**

The role of ^18^F-flurodeoxyglucose (^18^F-FDG) positron emission tomography–computed tomography (PET/CT) in colorectal cancer (CRC) remains unclear. This study aimed to explore the association of the maximum standardized uptake value (SUVmax), a parameter of ^18^F-FDG PET/CT, with KRAS mutation, the Ki-67 index, and survival in patients with CRC.

**Methods:**

Data of 66 patients with CRC who underwent ^18^F-FDG PET/CT was retrospectively collected in our center. The clinical significance of the SUVmax in CRC and the association of the SUVmax with KRAS mutation and the Ki-67 index were determined. A meta-analysis was conducted by a systematic search of PubMed, Web of Science, and CNKI databases, and the data from published articles were combined with that of our study. The association of the SUVmax with KRAS mutation and the Ki-67 index was determined using the odds ratio to estimate the pooled results. The hazard ratio was used to quantitatively evaluate the prognosis of the SUVmax in CRC.

**Results:**

By analyzing the data of 66 patients with CRC, the SUVmax was found not to be related to the tumor-node-metastasis stage, clinical stage, sex, and KRAS mutation but was related to the tumor location and nerve invasion. The SUVmax had no significant correlation with the tumor biomarkers and the Ki-67 index. Data of 17 studies indicated that the SUVmax was significantly increased in the mutated type compared with the wild type of KRAS in CRC; four studies showed that there was no remarkable difference between patients with a high and low Ki-67 index score regarding the SUVmax. Twelve studies revealed that the SUVmax had no significant association with overall survival and disease-free survival in CRC patients.

**Conclusions:**

Based on the combined data, this study demonstrated that the SUVmax of ^18^F-FDG PET/CT was different between colon and rectal cancers and associated with KRAS mutation but not the Ki-67 index; there was no significant association between the SUVmax and survival of patients with CRC.

## Introduction

According to the GLOBOCAN 2020 reports, colorectal cancer (CRC) is the third most common malignancy and the second most common cause of cancer-related deaths worldwide (1). The outcome of patients with CRC has greatly improved with the advancement of multimodality treatment. However, the prognosis remains poor for patients at late stages (clinical stages III and IV) since these patients present tumor metastasis and poor response to treatment (2, 3). Unfortunately, many patients with CRC are diagnosed at the advanced stage. Therefore, the early detection and prediction of CRC is still a challenge for physicians (4). Currently, the most commonly used indicator in predicting the survival of patients is the tumor-node-metastasis (TNM) stage. Besides, some gene mutations or endogenous proteins are also reported to be associated with the prognosis of patients with CRC, such as KRAS mutation and the Ki-67 index (5, 6). Moreover, the non-invasive imaging biomarkers of cellular proliferation represent a great promising prospect for precision medicine in CRC (7).

To date, ^18^F-flurodeoxyglucose (^18^F-FDG) positron emission tomography (PET) computed tomography (CT) has been used for the diagnosis, monitoring treatment response, surveillance of local recurrence, and prognosis for CRC (8, 9). ^18^F-FDG PET is a qualitative and quantitative method used to evaluate tumor development (10–12). The standardized uptake value (SUV) is one of the important semi-quantitative parameters, which is used to assess the degree of ^18^F-FDG accumulation. Previously, several studies reported that the maximum SUV (SUVmax) was associated with the lymph node metastasis of CRC, and served as a potential predictor of survival in patients with CRC, indicating its promising value (13, 14). Furthermore, studies suggested that the SUVmax was greatly increased in patients with KRAS mutations, which is particularly crucial to the therapeutic strategy (15).

Despite several studies showing the association between the SUVmax and KRAS mutation and prognostic value in CRC, inconsistent results have been found in some studies (16, 17). Generally, the causes of the disparity in the results can be explained by the small sample size. Besides, Ki-67 was described to be correlated with the proliferative capacity, invasive potential, and prognosis of CRC in two studies (18, 19). However, the number of the patients was small, and the results still need to be further validated. Therefore, this study aimed to explore the role of the SUVmax of ^18^F-FDG PET/CT in CRC by analyzing the association of the SUVmax with the clinical features of CRC, KRAS mutation, and the Ki-67 index. We also performed a meta-analysis by combining data from previous studies to confirm our results and explored the prognostic value of the SUVmax in patients with CRC.

## Patients and Methods

### Study Population

Data of patients with CRC who underwent ^18^F-FDG-PET/CT scans before tumor resection at Guangxi Medical University Cancer Hospital was retrospectively collected between May 2018 and December 2021. The inclusion criteria were as follows: the diagnosis of CRC confirmed by histological examination of surgical specimens and patients who did not undergo chemotherapy and/or radiotherapy before the examination. Patients with autoimmune diseases, and severe or major organ failure were excluded since these diseases could affect the metabolism of FDG (20, 21), although no clear evidence showed that they have a correlation with CRC. Finally, a total of 66 patients with CRC were included in this study, including 60 with colon cancer and 6 with rectal cancer. There were 25 patients with distant metastasis, and there were 40 male and 26 female patients, and the average age of the patients was 55.5 years. The most common histological type of CRC was adenocarcinoma. Information on the clinical features of patients, age, sex, TNM stage, tumor biomarkers, blood routine examination findings, KRAS mutation, and Ki-67 index scores were extracted. The TNM stage was defined based on the American Joint Committee on Cancer criteria 8th edition (22). The study protocol was approved by the Institutional Review Board of Guangxi Medical University Cancer Hospital, and all patients provided written consent.

### ^18^F-FDG PET/CT Imaging and Analysis

The ^18^F-FDG PET/CT protocol and interpretation were described in a previous study (23). All patients underwent whole-body FDG PET using a GE Discovery 710 PET/CT scanner (General Electric Medical Systems, GEMS, USA). The tube voltage is set as 120 kV, and the tube current is 200 mAs. The slice thickness is 3.75 mm. PET collection uses 3D mode PET scanning, 2.5 min/bed, and generally scans six to eight beds. Image recombination reconstructs images using the ordered subset maximum expectation method. Patients fasted for at least 6 h, and serum glucose levels were confirmed to be less than 180 mg/dl prior to ^18^FDG administration. PET/CT images were obtained 60 min after the administration of 370–450 MBq of ^18^FDG. Oral contrast material was used in all patients for a good visualization of the intestinal lumens. CT scans that were used for attenuation correction were performed just before PET acquisitions. PET data were acquired from the top of the skull to the upper thigh with the arms in supine position. The PET/CT images were transferred to GE Xeleris workstation. Two physicians who were blinded to the diagnosis of diseases reviewed and assessed the images. The SUVmax for body weight was calculated by drawing a region of interest on the attenuation-corrected transaxial FDG PET images. If there were multiple lesions, we only selected the highest SUVmax in the analysis.

### KRAS Mutation Analysis

The detection of KRAS mutation was conducted in our previous study (24). In brief, the DNA was extracted from formalin-fixed, paraffin-embedded tumor tissue sections by the QIAamp FFPE Tissue Kit (QIAGEN KK). Mutations in KRAS codons 12 and 13 in exon 2 were detected using amplification refractory mutation system polymerase chain reaction methods. Amplicons were detected using capillary electrophoresis on an ABI 3130xl Genetic Analyzer (Applied Biosystems/Life Technologies, Grand Island, NY) and analyzed using GeneMapper Software (Applied Biosystems/Life Technologies) according to the manufacturer’s instruction.

### Immunohistochemical Staining for Ki-67

IHC assays were performed on formalin-fixed, paraffin-embedded, and 4-μm-thick sections of tissue samples using primary antibodies against Ki-67 primary antibody (Novocastra, NCL-MM1, 1/100). The Ki-67 labeling index score was calculated in five randomly selected areas in each tumor sample as the number of Ki-67 positive cells/total counted at 400× magnification.

### Statistical Analysis

The continuous data (SUVmax value) are expressed as mean and standard deviation (SD), median, distribution frequencies, and percentages, when appropriate. The Mann–Whitney *U*-test or Student’s *t*-test was employed to compare continuous data between the two groups, when appropriate. The analysis of variance method (ANOVA) was applied to compare the continuous variables among the three groups. Normalization of data distribution was evaluated using Kolmogorov–Smirnov test. For variables that were not normally distributed, comparison was performed using Mann–Whitney *U*-tests. Correlation among SUVmax, tumor biomarkers, and Ki-67 was analyzed using the Pearson analysis. All analyses were two sided, and statistical significance was set at a *p*-value less than 0.05. Statistical analyses were conducted using the R Software (version 3.6.3).

### Meta-Analysis for the Data

The meta-analysis was performed in accordance with the guidelines of the Preferred Reporting Items for Systematic Review and Meta-analyses statement. Briefly, the relevant articles were retrieved and assessed from the databases (Web of Science, PubMed, and Chinese National Knowledge Infrastructure) before January 2021, using “colorectal cancer”, “FDG”, “flurodeoxyglucose”, “PET/CT”, “KRAS”, “Ki-67”, or “survival” as search terms. Based on certain criteria, data (the author’s name, country, number of patients, SUVmax, KRAS mutation, Ki-67 index, follow-up, and hazard ratio [HR] for survival) were extracted from these articles. The association of the SUVmax with KRAS mutation and the Ki-67 index was determined using the odds ratio (OR) to estimate the pooled results. The HRs and its 95% CI were used to quantitatively evaluate the prognosis of the SUVmax in CRC. The random-effects model (DerSimonian–Laird method) was used to combine the data if there was significant heterogeneity across the studies; otherwise, the fixed-effects model (Mantel–Haenszel method) was conducted. R language (version 3.6.3) with “meta” package was used to conduct the meta-analysis. Statistical significance was set at *p*-values less than 0.05.

## Results

### Clinical Characteristics of Patients With CRC

**Figure 1:**
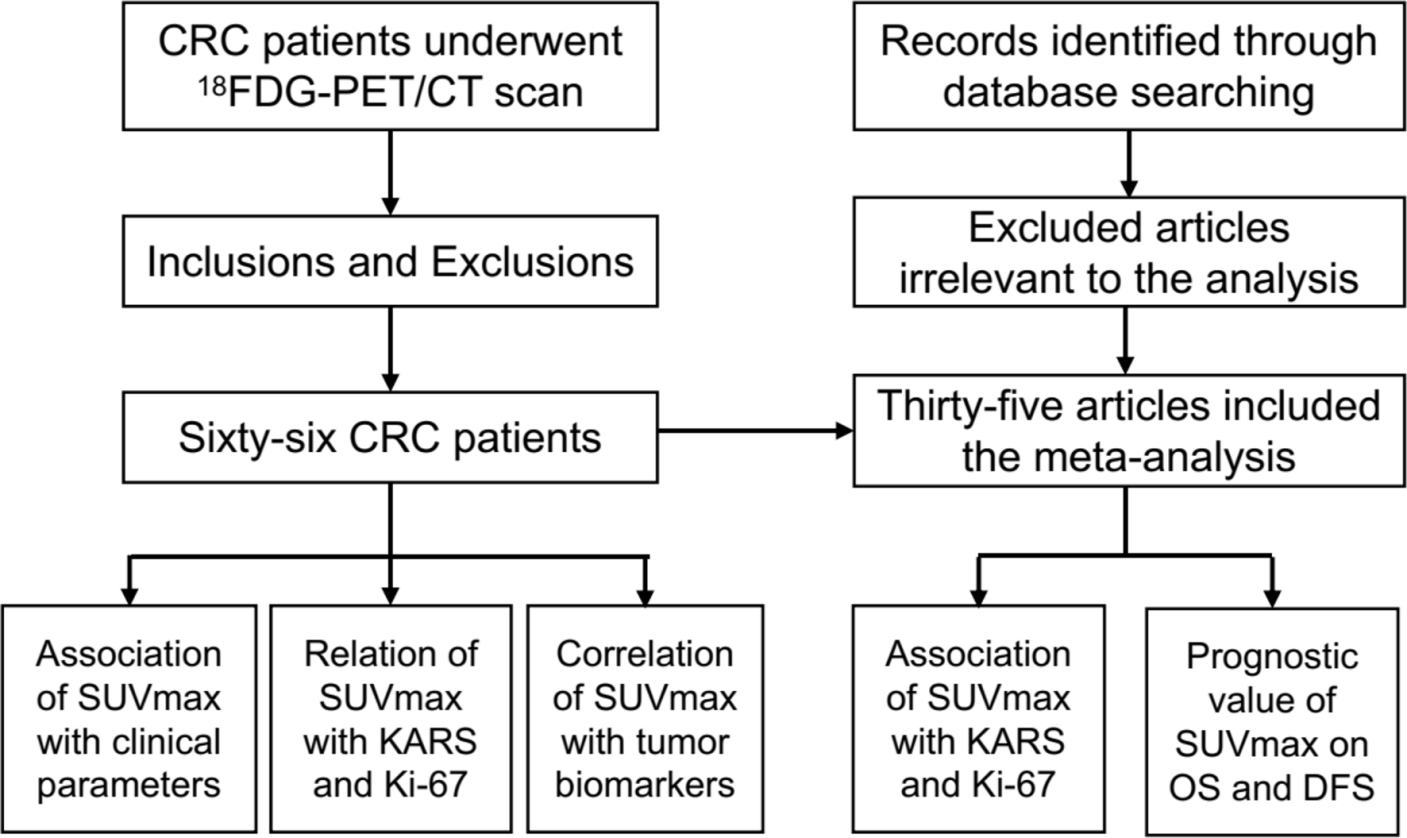
The flow chart of the present study.

The flow chart of the present study is presented in **Figure 1**. Based on the inclusion criteria, the present study finally included 66 CRC patients, namely, 60 colon cancers and 6 rectal cancers, and 25 patients with tumor distant metastasis; there were 40 male and 26 female patients, and the average age of the patients was 55.5 years old. All the histology type of CRC was adenocarcinoma. The SUVmax was collected from the primary lesion of CRC, and the mean SUVmax of the patients was 14.75. The details of the patients are listed in **Table 1**.

**Table 1:** Clinical characteristic of CRC patients.

Variables	Value
Age (years)	55.5 ± 13.3
Sex	
Male	40 (60.6%)
Female	26 (39.4%)
Location	
Right colon	14 (21.2%)
Left colon	46 (69.7%)
Rectal	6 (9.09%)
Vascular embolism	
No	52 (78.8%)
Yes	14 (21.2%)
Nerve invasive	
No	41 (62.1%)
Yes	25 (37.9%)
Differentiation degree	
Low	9 (13.6%)
Middle	50 (75.8%)
High	1 (1.52%)
NA	6 (9.09%)
T stage	
T1	6 (9.09%)
T2	9 (13.6%)
T3	13 (19.7%)
T4	38 (57.6%)
N stage	
N0	31 (47.0%)
N1	17 (25.8%)
N2	13 (19.7%)
Nx	5 (7.58%)
M stage	
M0	40 (60.6%)
M1	25 (37.9%)
Mx	1 (1.52%)
Clinical stage	
I	5 (7.58%)
II	20 (30.3%)
III	17 (25.8%)
IV	24 (36.4%)
White blood cells (×10^9^/L)	6.53 ± 1.52
Red blood cells (×10^9^/L)	4.27 ± 0.70
Platelet (×10^9^/L)	281 ± 115
Leukocyte (×10^9^/L)	4.08 ± 1.41
Lymphocyte (×10^9^/L)	1.70 ± 0.61
CA125 (ng/ml)	24.4 ± 45.2
CA153 (U/ml)	10.5 ± 4.33
CA199 (U/ml)	134 ± 316
CRP (mg/L)	37.9 ± 60.0
CEA (ng/ml)	27.5 ± 66.7
KRAS status	
Mutated type	18 (27.3%)
Wild type	14 (21.2%)
NA	34 (51.5%)
SUVmax	14.6 ± 8.69
Ki-67(%)	0.69 ± 0.17

### Clinical Significance of the SUVmax in Patients With CRC

**Figure 2:**
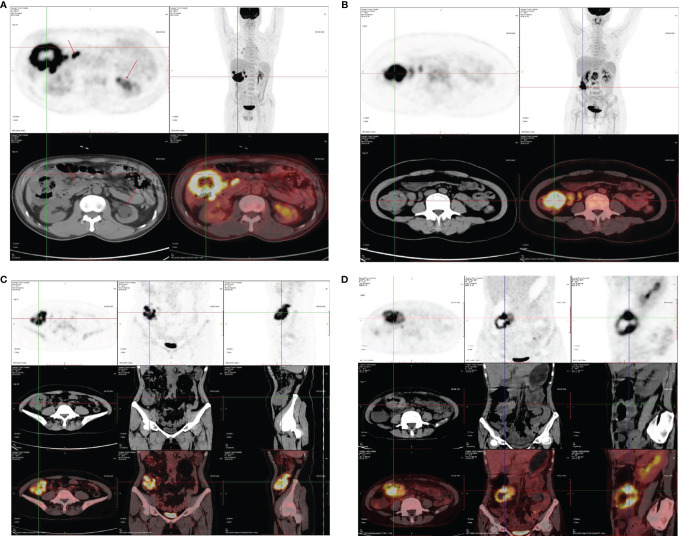
Representative images ^18^FDG-PET/CT scan on CRC patients. **(A)** Ascending colon, stage IV, SUVmax: 34.0. **(B)** Ascending colon, stage III, SUVmax: 19.9. **(C)** Transverse colon, stage II, SUVmax: 21.3. **(D)** Ascending colon, stage II, SUVmax: 26.1.

**Figure 3:**
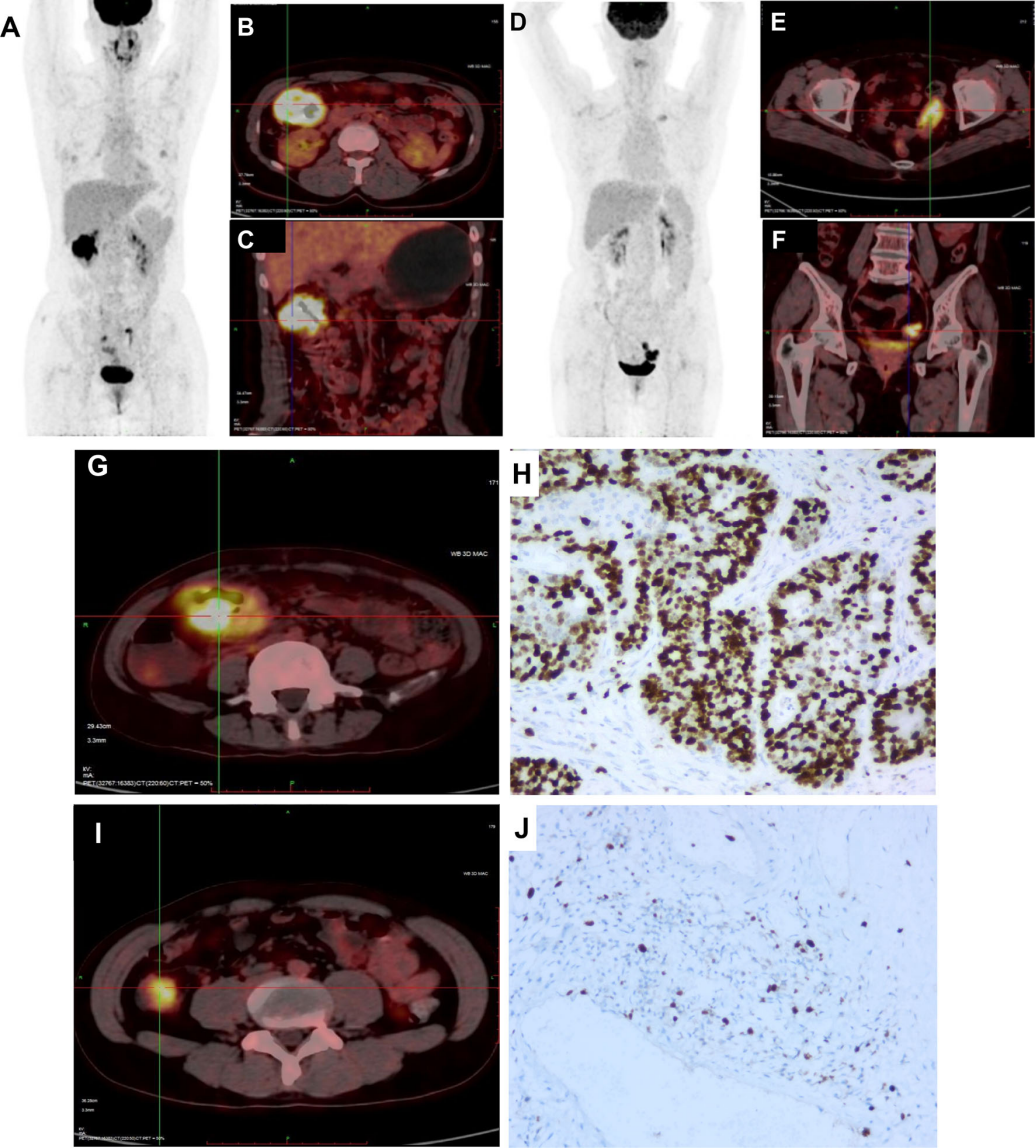
Representative images of KRAS mutation of ^18^FDG-PET/CT scan and Ki-67 index in CRC patients. **(A)** 18FDG-PET/CT scans image of CRC patient with mutated-type KRAS. **(B, C)** 18FDG-PET/CT scans showed intense accumulation of 18FDG in the hepatic flexure of colon (SUV: 20.0). **(D)** 18FDG-PET/CT scans image of CRC patient with wild-type KRAS. **(E, F)** 18FDG-PET/CT scans showed intense accumulation of 18FDG in the sigmoid colon (SUV: 12.4). **(G)** 18FDG was accumulated in the sigmoid colon (SUV: 21.3). **(H)** Maximum IHC expressions are 77.8% for Ki-67. **(I)** 18FDG was accumulated in the sigmoid colon (SUV: 12.4). **(J)** Maximum IHC expressions are 22.7% for Ki-67.

The representative images of ^18^F-FDG -PET/CT scans on CRC patients in different stage are listed in **Figure 2**. The representative images of KRAS mutation of ^18^F-FDG-PET/CT scans and the images of Ki-67 indices in patients with CRC are shown in **Figure 3**. As shown in **Table 2**, the SUVmax had no significant associations with the TNM stage, clinical stage, vascular embolism, sex, differentiation degree, and KRAS mutation but was related to the tumor location (*p* = 0.003) and nerve invasion (*p* = 0.007). When the data of rectal cancer were removed, the results for colon cancer were still similar to the overall results.

**Table 2:** Comparison of SUVmax in clinical parameters of CRC patients.

Variables	Number	Value	*p*-value
Sex			
Male	40	14.76 ± 10	0.825
Female	26	14.31 ± 6.37	
Location			
Right colon	14	18.61 ± 11.03	0.001
Left colon	46	14.77 ± 7.28	
Rectal	6	3.68 ± 1.23	
Vascular embolism			
No	52	14.79 ± 9.46	0.604
Yes	14	13.8 ± 5.1	
Nerve invasive			
No	41	11.82 ± 6.13	0.011
Yes	25	15.07 ± 9.04	
Differentiation degree			
High	1	1.6	0.205
Low	9	11.82 ± 6.13	
Middle	50	15.07 ± 9.04	
T stage			
T1	6	9.02 ± 4.92	0.384
T2	9	15.64 ± 5.65	
T3	13	16.29 ± 6.6	
T4	38	14.62 ± 10.11	
N stage			
N0	31	14.27 ± 8.03	0.489
N1	17	15.17 ± 11.47	
N2	13	12.62 ± 6.82	
Nx	5	19.64 ± 5.7	
M stage			
M0	40	13.42 ± 5.94	0.377
M1	25	16.22 ± 11.88	
Mx	1	19.9	
Clinical stage			
I	5	11.28 ± 5.5	0.321
II	20	15.71 ± 6.3	
III	17	11.89 ± 4.9	
IV	24	16.23 ± 12.14	
KRAS status			
Mutate-type	18	17.59 ± 8.67	0.629
Wild-type	14	15.77 ± 11.65	

We next examined the correlation of the SUVmax with tumor biomarkers, including the CEA, CA125, CA153, and CA199; no significant correlation was observed between the SUVmax and tumor biomarkers (*p* > 0.05). Regarding the correlation of the SUVmax with the routine blood test, we found that the SUVmax was remarkably correlated to the platelet levels (*p* < 0.05). The Ki-67 index, another indicator that represents the cellular proliferation in tumor, showed no significant correlation with the SUVmax (*p* > 0.05).

### Meta-Analysis for the Association of the SUVmax With the KRAS Status in CRC

**Figure 4:**
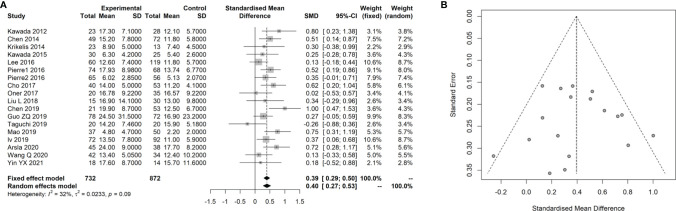
Meta-analysis for the association of SUVmax with KRAS status in CRC cancer. **(A)** Forest plot for the association of SUVmax with KRAS status. **(B)** Funnel plot for the publication bias.

Sixteen studies (14–17, 25–36) with 1,727 patients with CRC that evaluated the association of the SUVmax with the KRAS status in CRC were included the meta-analysis. The details of included studies are listed in **Table 3**. All studies were retrospective and most patients underwent treatment before the PET-CT examination. The age of patients ranged from 55 to 68 years. By combining these data with the present study, we found that the SUVmax in patients with a mutated type of KRAS was significantly increased compared with those with KRAS wild-type [SMD: 0.39, 95% confidence interval (CI): 0.29–0.50; Mantel–Haenszel method, *I* ^2^: 32%, *p* heterogeneity: 0.09; **Figure 4A**]. There was no significant publication bias across the 17 studies (Egger test: *p* = 0.950; **Figure 4B**).

**Table 3:** Characteristics of studies analyzed the association of SUVmax with KRAS and Ki-67 in colorectal cancer.

Authors	Year	Country	Age	No. Patient	Design	Tumor type	Treatment
**KRAS mutation**							
Kawada et al. (25)	2012	Japan	68	51	R	CRC	No
Chen et al. (26)	2014	China	59	121	R	CRC	Yes
Krikelis et al. (16)	2014	Greece	60	44	R	CRC	No
Kawada et al. (27)	2015	Japan	65	55	R	CRC	No
Lee et al. (29)	2016	Korea	60	179	R	CRC	No
Lovinfosse et al. (17)	2016	Belgium	66	151	R	CRC	No
Lovinfosse et al. (17)	2016	Belgium	66	151	R	CRC	No
Cho et al. (30)	2017	Korea	60	184	R	CRC	NA
Oner et al. (33)	2017	Turkey	55	55	R	CRC	No
Liu et al. (36)	2018	China	61	45	R	CRC	No
Chen et al. (28)	2019	China	58	74	R	CRC	No
Guo et al. (31, 37)	2019	China	62	132	R	CRC	No
Taguchi et al. (34)	2019	Japan	67	40	R	CRC	No
Mao et al. (32)	2019	China	61	87	R	CRC	No
Lv et al. (14)	2019	China	56	164	R	CRC	No
Arsla et al. (15)	2020	Turkey	65	83	R	CRC	No
Wang et al. (35)	2020	China	66	76	R	CRC	No
**Ki-67 index**							
Fukuda et al. (18)	2019	Japan	68	18	P	CRC	NO
Nakajo et al. (19)	2014	Japan	64	30	P	CRC	YES
Song1 (38)	2019	China	59	358	R	Colon	NO
Song 2 (38)	2019	China	59	358	R	Rectal	NO

NA, not available; P, prospective; R, retrospective.

### Meta-Analysis for the Association of the SUVmax With the Ki-67 Index in CRC

**Figure 5:**
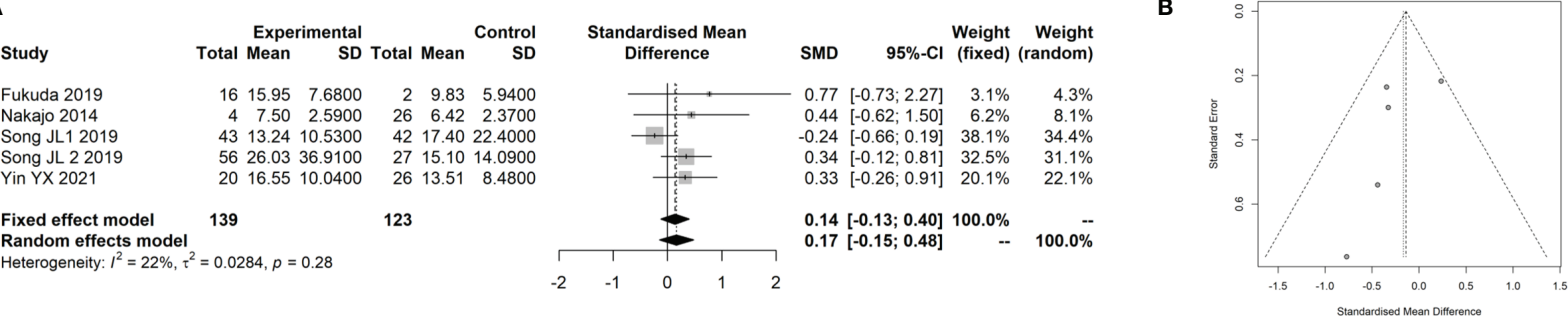
Meta-analysis for the association of SUVmax with Ki-67 index in CRC cancer. **(A)** Forest plot for the association of SUVmax with Ki-67 index. **(B)** Funnel plot for the publication bias.

Three studies (18, 19, 38) with 250 patients with CRC that evaluated the association of the SUVmax with the Ki-67 index in CRC were included in the meta-analysis. The details of included studies are listed in **Table 3**. Two of the studies were prospectively designed, and the age of patients ranged from 56 to 68 years. We divided the patients into two groups using 75% of the Ki-67 index score as cutoff. By pooling these data with our data, we found that there was no remarkable difference between patients at a high and low Ki-67 index score regarding the SUVmax (SMD: 0.14, 95% CI: −0.13–0.40; Mantel–Haenszel method, *I* ^2^: 22%, *p* heterogeneity: 0.28; **Figure 5A**). There was no significant publication bias found in the four studies (Egger test: *p* = 0.293; **Figure 5B**).

### Meta-Analysis for the Prognostic Value of the SUVmax in CRC

**Figure 6:**
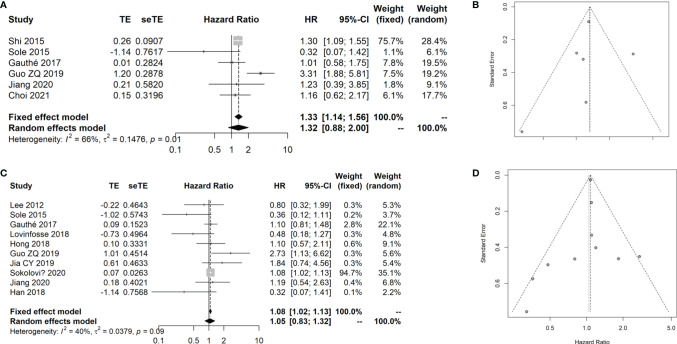
Meta-analysis for the prognostic value of SUVmax in CRC cancer. **(A)** Forest plot for the association of SUVmax with OS. **(B)** Funnel plot for the publication bias of OS data. **(C)** Forest plot for the association of SUVmax with DFS/PFS. **(D)** Funnel plot for the publication bias of DFS/PFS data.

Sixteen studies (13, 37, 39–48) with 1,325 patients with CRC that evaluated the prognostic value of the SUVmax in patients with CRC were included in the meta-analysis. The details of included studies are listed in **Table 4**. Two studies were prospectively designed, and the age of patients ranged from 57 to 66 years. Two studies provided the survival data from univariate instead of multivariate Cox regression. In order to analyze the prognostic value of the SUVmax in different survival data types, we divided the patients into two groups based on the survival data type, including overall survival (OS) (8, 31, 32, 35, 37, 40) and disease-free survival (DFS) or progression-free survival (PFS) (37, 40–48). By combining these data, we found that the SUVmax was not associated with OS of patients with CRC; however, there was significant heterogeneity across the studies (HR: 1.32, 95% CI: 0.88–2.00; Random effect method, *I* ^2^: 66%, *p* heterogeneity: 0.01; **Figure 6A**). No significant publication bias was observed among the studies (Egger test: *p* = 0.848; **Figure 6B**).

**Table 4:** Characteristics of studies analyzed the prognosis of SUVmax in colorectal cancer.

Authors	Year	Age	Country	No. Patient	Design	Tumor type	Treatment	Follow up	Cutoff	Type
Lee et al. (44)	2012	60	Korea	163	R	CRC	Yes	12 m	8.6	DFS
Sole et al. (47)	2015	62	Spain	37	R	Rectal	Yes	74 m	8.7	DFS
Gauthé et al. (48)	2017	63	France	75	R	Rectal	Yes	51 m	18	PFS
Lovinfosse et al. (45)	2018	66	Belgium	86	R	Rectal	Yes	60 m	NA	DFS
Hong et al. (41)	2018	60	USA	30	P	Rectal	Yes	30 m	NA	DFS
Guo et al. (31, 37)	2019	62	China	132	R	CRC	No	72 m	19.36	DFS
Jia et al. (42)	2019	57	China	73	R	CRC	no	48 m	14.80	DFS
Sokolović et al. (46)	2020	62.2	B&H	70	R	CRC	No	12 m	4.1	DFS
Jiang et al. (43)	2020	60	China	65	R	CRC	No	36 m	3.5	DFS
Han et al. (40)	2018	62	Korea	96	R	CRC	No	60 m	17.60	PFS
Shi et al. (13)	2015	60	China	107	P	CRC	No	60 m	11.85	OS
Sole et al. (47)	2015	62	Spain	37	R	Rectal	Yes	74 m	8.7	OS
Gauthé et al. (48)	2017	63	France	75	R	Rectal	Yes	51 m	18	OS
Guo et al. (31, 37)	2019	62	China	132	R	CRC	No	35 m	19.36	OS
Jiang et al. (43)	2020	60	China	65	R	CRC	No	36 m	3.5	OS
Choi et al. (39)	2021	59	Korea	82	R	CRC	No	60 m	8.7	OS

B&H, Bosnia and Herzegovina; P, prospective; R, retrospective; OS, overall survival; DFS, Disease-free survival; NA, not available.

The results showed that the SUVmax has no remarkable association with the DFS/PFS of patients with CRC (HR: 1.08, 95% CI: 1.02–1.13; Random effect method, *I* ^2^: 40%, *p* heterogeneity: 0.09; **Figure 6C**). No significant publication bias was observed among the studies (Egger test: *p* = 0.856; **Figure 6D**).

## Discussion

As one of the important parameters of ^18^F-FDG PET/CT, the SUVmax is able to measure the activity of tissue metabolism, which is strongly correlated with cell division and proliferation (49). Thus, the SUVmax is considered to sensitively predict the biological behavior of tumors that is influenced by various clinical factors (50). At present, the SUVmax has been widely applied to assess the malignant lesions from benign ones and could act as a critical indicator to predict the prognosis of patients with cancers. Using the data from our center, the present study found that the SUVmax was associated with the location of tumors and was remarkably increased in colon cancer compared with that in rectal cancer, which is similar to the findings of previous reports (35, 44). We also found that the SUVmax was associated with nerve invasion of CRC, which was not reported previously. However, the results did not show an association between the SUVmax and TNM and clinical stages of CRC. Moreover, no significant association was found between the SUVmax and KRAS mutation and the Ki-67 index. We conducted a meta-analysis due to the small sample size of the present study, by combining the previous published studies in order to achieve a robustness of results. In the meta-analysis, we found that the SUVmax was associated with the KRAS mutation status but not with OS or DFS of patients with CRC. The meta-analysis results also confirmed that there was no association between the SUVmax and Ki-67 index in patients with CRC. Considering the increasing application of ^18^F-FDG PET/CT in the clinical setting, our results provided important information for helping to make appropriate treatment strategy for the patients.

A previous study has shown that some PET parameters, such as SUVmax, SUVmean, and MTV, can guide the differentiation of benign, premalignant, and malignant lesions with incidentally detected foci of colorectal uptake [32092771]. There was a study that showed that focal colorectal ^18^F-FDG uptake on PET/CT is associated with a high risk of advanced colorectal neoplasia and is affecting subsequent patient management (51). These results suggested that various histopathological degrees might affect the FDG uptake value. In addition to the most commonly used ^18^F-FDG, ^68^Ga-fibroblast-activation-protein inhibitors (FAPIs) and ^177^Lu-prostate-specific membrane antigen (PSMA)-617 are two other radioisotopes used in PET/CT determination. Kratochwil et al. (52) evaluated ^68^Ga-FAPI PET/CT uptake in 28 different kinds of cancer and showed that the highest average SUVmax was found in sarcoma, esophageal, breast, cholangiocarcinoma, and lung cancer. The lowest ^68^Ga-FAPI uptake was observed in pheochromocytoma, renal cell, differentiated thyroid, adenoid cystic, and gastric cancer. Cuda et al. (53) performed ^68^Ga-PSMA-11 PET/CT imaging on 10 patients with metastatic CRC to assess metastasis avidity, and they showed that none of the patients exhibited tumor avidity sufficient to be considered for ^177^Lu-PSMA-617 PRRT. These results indicated that ^68^Ga-PSMA-11 and ^177^Lu-PSMA-617 PRRT might be an important alternative to the ^18^F-FDG.

In the present study, we analyzed the SUVmax data and clinical features data of 66 CRC patients, which included 60 colon cancers and six rectal cancers. A previous study has reported that SUVmax was associated with the lymph node metastasis and clinical stage of CRC (43). However, the results of the 66 CRC failed to show significant differences in the SUVmax regarding lymph metastasis and distant metastasis; after the data of rectal cancer were removed (only six cases), the differences in the SUVmax slightly increased in these two parameters. Therefore, the difference of SUVmax between colon and rectal cancers should be made cautiously.

The association of ^18^FDG accumulation with KRAS mutations in CRC was explored previously, but the results were inconsistent. The results of the 66 CRC suggested little association between the SUVmax and KRAS, which was consistent with what Kawada et al. (27) reported but in contrast to the results of Chen et al. (26). We speculated that the reasons might be that, in addition to the small sample size of each study, the doubtful locoregional disease, such as polyps, and precancerous lesions might affect the ^18^FDG accumulation. The common tumor biomarkers, including CEA, CA125, CA153, and CA199, have been used to evaluate the CRC development, treatment effect, or prognosis in clinical setting. Regarding the correlations between SUVmax and tumor biomarkers, Sokolović et al. (46) found that SUVmax was associated with CEA levels, but not associated with CA199. Our results failed to show significant correlation between SUVmax and CEA, CA125, CA153, or CA199 levels, which was partly consistent with previously reported data (46).

With respect to the Ki-67 index, we did not find a significant correlation with SUVmax, which was in agreement with previous reports (18, 38). Taken together, the results from the 66 CRC patients showed that SUVmax has little associations with the clinical features of CRC, KRAS mutation, tumor biomarkers, and Ki-67 index. However, considering the fewer cases in those studies, this association needs to be confirmed by a larger study or by adding more samples to improve the reliability of the results. In addition, due to the lack of survival data, the prognostic value of SUVmax still needs to be determined. Therefore, we subsequently performed a meta-analysis to further validate the clinical significance of SUVmax in CRC patients.

Since some of the epidemiological indicators, such as age and gender, were associated with the development of CRC, they could influence the association of SUVmax with the CRC. In our data, we did not observe differences between age and gender with the SUVmax, which were similar to the previous studies, demonstrating that these epidemiological indicators might not affect the association of SUVmax with the CRC.

A previous study has indicated that different differentiation of CRC was associated with the KRAS genes mutation (54); thus the differentiation of CRC might also lead to the difference in SUVmax in these cases. However, in our data, we showed no significant difference among the different differentiation of CRC, which was similar to the results of Lee et al. (44), but contrary to the Jiang et al. (43) report; these inconsistent results might be due to the small sample size or other reasons, suggesting that the exact impact of differentiation of CRC on the association with SUVmax still needs to be further validated in a larger cohort.

KRAS mutation occurs in approximately 40% of CRC patients, which is a crucial biomarker for the selection of patients who are suitable for the epidermal growth factor receptor therapy (55). Previously, several studies have indicated that the SUVmax was elevated in the mutated-type KRAS compared with the wild-type KRAS patients with CRC. For example, Chen et al. (19) analyzed the data from 121 patients with CRC and found that the SUVmax was significantly associated with the KRAS mutation status, and similar results were found in the report by Kawada et al. (18). However, inconsistent results were also reported in the study conducted by Krikelis et al. (11). Recently, a study conducted a meta-analysis using the data from nine studies with 804 patients, and the results observed low sensitivity and specificity of ^18^F-FDG for the prediction of KRAS mutation in patients with CRC. However, this meta-analysis did not specifically analyze the SUVmax or other parameters in PET/CT inspection, which reduced the reliability of the results. In the present study, we included studies with patients with CRC and the results demonstrated that the SUVmax in patients with mutated-type KRAS was significantly increased compared with those with wild type, suggesting that there is a significant association between the SUVmax and KRAS mutation.

Regarding the Ki-67, it is mainly expressed in proliferating cells from the G1 to the M-phase of the cell cycle. Previous studies reported that a high expression of Ki-67 was an independent good prognostic indicator in CRC (56, 57). The SUVmax and Ki-67 index are the direct markers of cellular proliferation; therefore, several studies explored the association of the SUVmax with Ki-67, but the sample size of the studies was small, which would undermine the association between them; thus, more studies are warranted to further determine their correlation. In this study, no correlation was found between Ki-67 expression and KRAS mutation, which was in agreement with the study by Petrowsky et al. (58). Since our study and previous studies included a small number of CRC cases, we performed a meta-analysis by combining the currently available studies and the data of our study in order to achieve reliable results. The results showed that the SUVmax has no significant difference between high or low Ki-67 expression using 75% as cutoff. Thus, the current results suggest that there is no significant association between the SUVmax and Ki-67 index based on the current lines of evidence. We speculate that at least two reasons could explain the little association between these two indicators. First, the sample size was small, which could not achieve statistical power. Second, in our study and the included studies, the different cutoff of Ki-67 might have also affected the association of these two indicators.

Preoperative prediction of patient’s prognosis could provide numerous benefits to the treatment selection and improve the treatment effect and quality of the patients. As a non-invasive imaging modality, whether the parameter in PET/CT inspection has a high prognostic value for patients with CRC is one of the most crucial issues. There were inconsistent results with respect to the association of SUVmax with the survival of patients with CRC. In this study, we retrieved data on the SUVmax to predict the survival of patients with CRC and found that the SUVmax has little association, on both OS and DFS, even in the subgroup analysis that divided the patients according to different countries or follow-up time. Thus, the present study did not support the prognostic value of the SUVmax in patients with CRC. However, we also noted that this result was obtained from a meta-analysis; the exact relation of SUVmax with CRC patient’s survival, especially a particular subtype of the cancer, needs to be validated in a large CRC cohort.

Despite several meta-analysis studies exploring the role of ^18^F-FDG PET/CT in CRC, including the diagnosis of lymph node metastasis, KRAS mutation, and prognostic value (59–61), none analyzed the association of the SUVmax with KRAS mutation, the Ki-67 index, and their prognostic value. In addition, this study included available studies and incorporated the data from our center, which can achieve reliable results compared with previous studies. Nevertheless, some limitations should be noted. First, the present study was retrospective in nature, and some of the included studies in the meta-analysis were also retrospective, which could lead to selection bias. Second, compared with some previous reports (28–30), the number of patients from our center was relatively small, especially those with rectal cancer; thus, the robustness of our results might be undermined. Third, in the meta-analysis results, some factors across the studies were heterogeneous, including the follow-up time, treatment strategy, and staging; thus, the possibility of bias caused by disease profiles should be noted. Therefore, our results still need to be further validated using a large cohort with a prospective design.

## Conclusions

By combining our data with previously published data, the present study demonstrated that the SUVmax of ^18^FDG-PET/CT is different in colon and rectal cancers and is associated with KRAS mutation but not with the Ki-67 index; there was no significant association between the SUVmax and survival of patients with CRC.

## Data Availability Statement

The raw data supporting the conclusions of this article will be made available by the authors, without undue reservation.

## Ethics Statement

The studies involving human participants were reviewed and approved by the Institutional Review Board of Guangxi Medical University Cancer Hospital. The patients/participants provided their written informed consent to participate in this study.

## Author Contributions

Study concept and design: Y-XY and B-LH. Collection and assembly of data: Y-XY and M-ZX. Performed the experiment: Y-XY, X-QL, and M-ZX. Data analysis and interpretation: B-LH, J-LL, and M-ZX. Manuscript writing and review: All authors. All authors contributed to the article and approved the submitted version.

## Funding

This study was partially supported by research funding from the National Natural Science Foundation (No. 81860417) and the Natural Science Foundation of Guangxi (No. 2018JJA140136).

## Conflict of Interest

The authors declare that the research was conducted in the absence of any commercial or financial relationships that could be construed as a potential conflict of interest.

## Publisher’s Note

All claims expressed in this article are solely those of the authors and do not necessarily represent those of their affiliated organizations, or those of the publisher, the editors and the reviewers. Any product that may be evaluated in this article, or claim that may be made by its manufacturer, is not guaranteed or endorsed by the publisher.
